# Factors affecting the technical efficiency of rural primary health care centers in Hamadan, Iran: data envelopment analysis and Tobit regression

**DOI:** 10.1186/s12962-020-00249-1

**Published:** 2020-11-23

**Authors:** Saeed Mohammadpour, Javad Javan-Noughabi, Ali Vafaee Najar, Moharram Zangeneh, Shaghayegh Yousefi, Mojtaba Nouhi, Reza Jahangiri

**Affiliations:** 1 Department of Health Economics, School of Health Management and Information Sciences, Iran University of Medical Sciences, Rashid Yasemi St. Vali-e Asr Ave, 19967-13883 Tehran, Iran; 2 Social Determinants of Health Research Center, Faculty of Health, Mashhad University of Medical Sciences, Daneshgah st. between 16-18, 91778-99191 Mashhad, Iran; 3 Department of Health Management, School of Health Management and Information Sciences, Iran University of Medical Sciences, Tehran, Iran; 4 Mashhad University of Medical Sciences, Mashhad, Iran; 5 Health Equity Research Center, Tehran University of Medical Sciences, Tehran, Iran

**Keywords:** Resource allocation, Efficiency, Organizational, Regression analysis, Primary health care, Rural health services, Iran

## Abstract

**Background:**

Studying and monitoring the efficiency of primary health care centers has a special place in the health system. Although studies have been conducted in the field of efficiency in Iran, few have focused on rural primary health care centers. In addition, previous studies have not used the child mortality rate and Behvarzes as input and output.

**Objective:**

The present study was conducted aimed to estimate the technical efficiency of rural primary health care centers and determinant factors in Hamadan using data envelopment analysis and Tobit regression.

**Methods:**

This is a Longitudinal study of rural primary health care centers in Hamadan province (2002–2016). Data Envelopment Analysis was employed to estimate technical efficiency of sampled health facilities while Panel Tobit Analysis was applied to predict factors associated with efficiency levels. The outputs were child mortality rate under 1 year of age and child mortality rate 1 year to 5 years of age. The input was Behvarzes (rural health workers).

**Results:**

The results of efficiency analysis showed that the average efficiency scores of the centers had a fluctuating trend during the period of the study, but the average performance scores generally decreased in 2016, as compared with 2002. The highest and lowest average performance scores were observed in 2003 (0.78) and 2013 (0.56), respectively. Number of physicians and rural primary healthcare centers per population had a positive statistically significant and the number of midwives and the total fertility per population had a negative statistically significant effect on efficiency.

**Conclusions:**

The findings suggest some level of wastage of health resources in primary health centers. Findings indicate a level of waste of health resources in primary health centers. Behvarz functions in providing primary care services can be considered in the reallocation and optimal use of available resources at the level of rural health centers.

## Introduction

Given the increasing growth in health care costs and the problems associated with financing the expenditures, policymakers have accepted that health care is not a mere social issue and should be addressed economically as well [1, 2]. According to the World Bank statistics and the World Health Organization reports, different countries spend an average of about 10% of their GDP on healthcare [3]. According to the latest reports, it is more than 8% in Iran [4–6]. In fact, the main threat for the health sector in most developing countries is the non-optimal utilization of resources and the inefficient role of resource management in solving problems [7, 8].

The primary health care (PHC) system are most important parts of every health system around the world [9]. Health centers and rural health houses are among the most important providers of primary health care. There has been a rapid growth in the number of health centers since the release of the Alma-Ata Declaration that had a key role in ensuring access to health for all people [10]. Iran government has made many efforts to establish a broad network of primary health care facilities, especially in rural areas, through rural health centers and health houses, aimed at reducing the gap between rural and urban services [11, 12].

According to most critics and stakeholders of the health sector in the county, the delivery of primary health care in Iran by Behvarz (local health worker), as a forefront of health especially in deprived and rural areas, has been one of the most important activities and achievements of the Iranian health system in the field of health. Health House is the most peripheral unit of service delivery in the health network system of the country that is located in rural areas and mainly based on Behvarz. Each health house may cover one or more villages depending on geographical conditions, especially transportation routes and population. The most important feature of health houses is the selection of Behvarzs according to the social conditions of the community [12, 13].

Mortality reduction, population control and family planning, vaccination for children, and maternal education can be attributed to the endless and admirable efforts of Behvarzs in rural areas [12, 13]. At the present, 29% of the country’s population lives in rural areas, so Behvarzs have a crucial role in the health system [12, 13].

Increasing demands for primary health services put primary health sector mangers in a situation to allocate resource more rational to reach better health outcomes within constraint budget. One of the way to deal with this challenge is identify level efficiency and focus to improve it within health houses in processes and outcomes dimensions [14, 15].

Unfortunately, in developing countries, including Iran, there is limited information on the effectiveness of primary health care centers, especially rural health houses. Various studies around the world as well as some of studies in Iran have examined the efficiency of primary health care centers [16–18]. However, it suffered from a crucial methodological flaw. They used some surrogate outcomes, while selecting a final outcome as a performance would be more reasonable to show level of efficiency in health houses. considering the nature of these centers, their diverse functional areas of activity, and their inputs and outputs, it can be stated that data envelopment analysis (DEA) is the best model for a comprehensive and clear evaluation of the centers [7].

The utilization of DEA not only helps to determine the relative efficiency and identify weaknesses of the organization, but also defines the organization's policy and approach towards promoting efficiency and productivity through presenting the desirability of performance indicators. It also defines efficient patterns, i.e. units that, as compared with other units, have more outputs while using a similar level of inputs or produce the same output using fewer inputs [19, 20].

Depending on the geographical location and demographic features of the target population, health houses may undertake different volumes of activity. Therefore, their performance is affected by the availability of various demographic and geographic resources and their performance should be evaluated.

Previous studies have examined various factors affecting efficiency, such as the following: distance from health centers, number of family members, religion, ethnidistrict, domestic livestock, durable household goods [21], access to safe drinking water, employee motivation [22], information and communication technology [23], socioeconomic variables, quality of care proxies, geographic location, site of centers, and the type of ownership on efficiency [24].

In fact, the measurement of the efficiency of rural primary health care centers can serve as a source of feedback for the managers. Due to the no study has been conducted to measure the efficiency of rural primary health care centers based on the performance of Behvarz and key health indicators in Iran and also the effective factors on the efficiency of these centers have not been identified. The aim of this study was estimate the technical efficiency of rural primary health care centers and determinant factors in Hamadan providence in Iran.

## Methods

This was a descriptive-analytical study using retrospective data from 2000 to 2016 among selected rural primary health care centers (PHCCs) in Hamadan province in Iran. Access to the required data during the study period was the most important criterion for selecting rural health centers. This study was conducted in two phases.

### Phase I

In the first phase data envelopment analysis (DEA) as a nonparametric method was used to evaluate the efficiency of rural primary health care centers.

#### Data envelopment analysis (DEA)

$$Max\;Effeciency_{p} = Max_{{u_{r} v_{i} }} \mathop \sum \limits_{r = 1}^{s} U_{r} Y_{rp} + U_{0} ,$$

$$S.t: \mathop \sum \limits_{r = 1}^{s} U_{r} Y_{rj} - \mathop \sum \limits_{i = 1}^{m} V_{i} X_{ij} + U_{0} \le 0;\forall_{i} ,$$

$$\sum V_{i} X_{ip} = 1 ,$$

$$U_{r} , V_{i} > 0;\forall_{r} ,\forall_{i} ,$$

where $U_{0}$ is the convexity constant and its sign determines the returns to scale. $U_{0} < 0$ indicates the increasing returns to scale, $U_{0} > 0$ indicates the decreasing returns to scale, and $U_{0} = 0$ indicates constant returns to scale. To measure efficiency according to the literature and access to available data, two outputs and one input were included. The outputs were child mortality rate under 1 year of age and child mortality rate 1 year to 5 years of age. The input was Behvarzes (rural health workers). Moreover, due to the fact that the outputs considered in the model are affected by many variables and the rural health centers cannot affect them, input-orientation approach and variable returns to scale (VRS) model were selected for data analysis. Data were collected from Health Center of the province as a management center of rural health centers in Hamadan province. Missing data were replaced by linear interpolation.

### Phase II

#### Tobit regression

The DEA efficiency scores were analyzed via regressing them against some characteristics of the PHC center to examine how these factors could affect the efficiency. The censored Tobit model was used since the dependent variable was censored at zero from below. In the regression models, where the range of change in the dependent variable is somehow restricted, the variables that take values in a limited range are defined as “censored” or “truncated” data. If the observations outside a certain range are excluded systematically from sample and completely lost, then they are called “truncated” data, and when observations do not provide any information about the dependent variable, but at least the independent variables could be observed, then they are called “censored” data [25]. If the observations obtained from the analysis of DEA are > 1, then they are not excluded from the sample, which is the same for truncated data. However, they cannot take their own values either and, thus, they are censored to 1 [26]. In this context, since the dependent variables that correspond to 1 can be observed, it has a censored structure.

Estimating a model with a censored dependent variable using ordinary least squares (OLS) method provides biased and inconsistent results in parametric estimations [27]. Furthermore, DEA scores have a relative efficiency index, rather than an absolute index, and the correlation between the efficiency scores make the OLS regression invalid [28]. Considering the mentioned reasons, Tobit regression, which is one of the limited dependent variable models that takes a censored structure into account, was used in the present study.

For parameter estimations, the Maximum Likelihood Estimation (MLE) method was used in the Tobit model. Since the parameters obtained through MLE are non-linear, the predictions of estimations were performed through iteration. Moreover, since it requires less time and fewer iterations as an iteration method, thus to offer other advantages, the Newton–Raphson method was utilized here [29].

The basic formula of panel Tobit used in this study was as follows:

1 $$y_{it}^{*} = \beta^{\prime}x_{it} + \varepsilon_{it} ,$$

2 $$y_{it} = \left\{ {\begin{array}{ll} {y_{it}^{*},\; if \; y_{it}^{*} < 1} \\ {1, \; otherwise } \\ \end{array} } \right.\quad i = 1, \ldots ,N\;{\text{and}}\;t = 1, \ldots ,T.$$

where subscript $i$ indicates the primary healthcare centers, subscript $t$ represents the time, $X_{it}$ is the explanatory variable in the dimension of 1 $\times$ k, and $\beta$ is the parameter vector on dimension of k $\times$ 1 [30].

We used efficiency score as dependent variable and run it against the independent variables. Four models were estimated via panel Tobit analysis. To select between pool methods and panel methods in Tobit model, panel data methods selected by the use of Chav test and observing F Limer statistics. Then, using the Hasman test and observing the chi-square statistics, the random effect model was selected against the fixed effect With including number of physicians, number of midwives and number of primary healthcare centers into the first model, the second model was obtained and with including total fertility into the model. The third model was generated by including total fertility, Number of physicians per population, Number of midwives per population and Number of primary healthcare centers per population. Number of physicians per population, Number of midwives per population and Number of primary healthcare centers per population were included in the fourth model. The likelihood ratio and p-value help to identify the model which fits significantly better than other models. In this study, the results of DEA were obtained using DEAP v2.1 and the results of Panel Tobit were obtained using STATA 12 software.

## Results

This study evaluated the efficiency of rural PHCCs at a provincial level. The productivity structure on the centers in eight cities from 2002 to 2016 was investigated through a two-stage analysis. DEA was used to assess the efficiency scores of DMUs, and Tobit regression was applied to evaluate the changes in total factor efficiency by years. The results obtained through applying DEA model and Panel Tobit are presented below.

Based on the results presented in Table 1 the highest average technical efficiency score was observed in 2003. The lowest technical efficiency was observed in Malayer district in 2007 and 2013 and in Hamedan district in 2016. The highest dispersion of technical efficiency score was observed in 2007. The assessment of the distribution of PHCCs based on the technical efficiency score showed that the rate of PHCCs with an efficiency score above the average in 2002 to 2005 was approximately 37%, and it even reached 50% in 2006; finally, in 2016, 62% of PHCCs had an efficiency score above the average.

**Table 1:** Technical efficiency values of eight primary health care centers

Cities	2002	2003	2004	2005	2006	2007	2008	2009	2010	2011	2012	2013	2014	2015	2016
Asadabad	0.937	1	0.895	0.81	1	1	1	1	0.936	0.876	0.726	0.63	0.68	0.523	0.63
Bahar	1	0.913	0.667	1	0.872	1	0.623	1	1	1	1	0.52	0.863	1	1
Toyserkan	0.993	1	1	1	1	0.9	0.875	0.85	0.97	0.783	0.912	1	1	0.923	0.82
Razan	0.64	0.672	0.373	0.62	0.744	0.615	0.567	0.81	0.617	0.551	0.486	0.52	0.458	0.494	0.43
Kaboodarahang	0.486	0.691	0.469	0..474	0.489	0.413	0.508	0.48	0.587	0.784	0.499	0.46	0.376	0.387	0.43
Malayer	0.458	0.406	0.339	0.44	0.584	0.313	0.497	0.39	0.35	0.592	0.43	0.33	0.601	0.55	0.46
Nahavand	0.564	0.733	0.448	0.63	0.613	0.353	0.595	0.36	0.495	0.614	0.685	0.63	0.689	0.665	0.6
Hamadan	0.585	0.491	0.581	0.41	0.474	0.399	0.559	0.66	0.393	0.532	0.438	0.41	0.487	0.487	0.33
Mean	0.708	0.738	0.597	0.700	0.722	0.624	0.653	0.694	0.669	0.717	0.647	0.562	0.644	0.629	0.587
SD	0.230	0.222	0.243	0.243	0.215	0.299	0.183	0.261	0.264	0.170	0.221	0.203	0.211	0.220	0.227

**Fig. 1:**
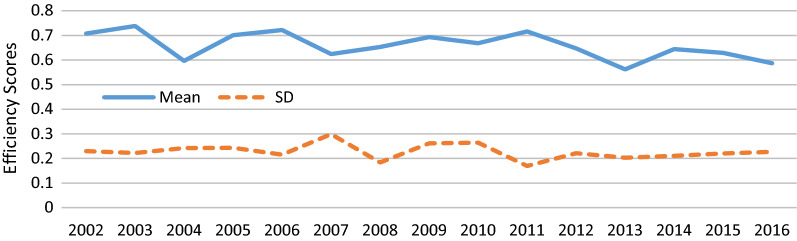
Mean and standard deviation of total efficiency score in time period

**Fig. 2:**
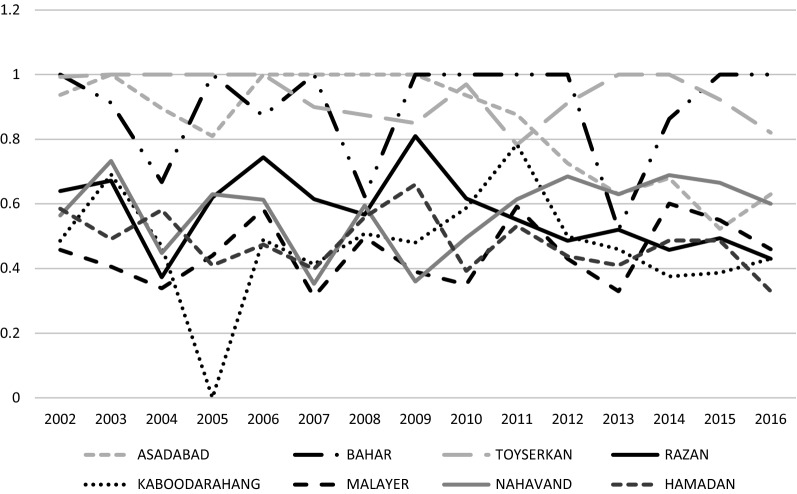
Mean of efficiency score based on primary health care centers in eight county of Hamadan province between 2002 and 2016

The assessment of the average technical efficiency scores of the PHCCs by years showed that the trend of efficiency scores changed and began to decrease in 2004. As the results showed the standard deviation of the technical efficiency scores in PHCCs was almost constant during the period of the study. As shown in Fig. 1, the mean of total efficiency of PHCCs has fluctuated a lot during the study period. Also, Mean of efficiency score based on primary health care centers in eight county of Hamadan province between 2002 and 2016 was shown in Fig. 2.

Taking into account the efficiency scores obtained from DEA as a dependent variable, Panel Tobit analysis was applied in the second stage of the study to examine the selected variables affecting the efficiency. The results of estimation through Panel Tobit Random Effects model are presented in Table 2.

**Table 2:** Results of random effects Tobit regressions

Variables	Model 1	Model 2	Model 3	Model 4
Number of physicians	0.0116734 (− 0.0069119)	0.0103544 (− 0.006829)*		
Number of midwives	− 0.0040891 (− 0.0057493)*	− 0.0030833 (− 0.0056645)**		
Number of primary healthcare centers	0.0084836 (− 0.0027772)	0.0111017 (− 0.0034194)		
Total fertility		− 0.0967611 (− 0.0642264)**	− 0.0234858 (− 0.0658905)**	
Number of physicians per population			1289.056 (− 0.00515)*	1313.28 (− 0.02221)**
Number of midwives per population			− 1118.806 (− 0.0937739)*	− 1111.251 (− 0.035053)**
Number of primary healthcare centers per population			850.818 (− 0.00750356)*	796.3428 (− 0.0052572)***
Log likelihood	− 26.635495	− 25.492471	− 30.20784	− 30.271497

* 0.05, ** 0.01, *** 0.001

Four models were estimated via Panel Tobit analysis. In the first model, the number of physicians, midwives, and rural primary healthcare centers in each district was included in the model, and a negative and significant relationship was observed between the number of midwives and efficiency but no significant relationship between the number of physicians and number of rural PHCCs. However, in the second model, with including the total number of fertility into the first model, it was observed that the number of physicians had a positive and significant relation with efficiency. Also number of midwifes and total fertility have a negative and statistically significant relationship with efficiency. In the third model, the number of physicians and midwives per population and the number of rural PHCCs and total fertility were included. Accordingly, in this model the number of physicians per population and the number of rural PHCCs had a positive statistically significant effect on efficiency, but the number of midwives and the total fertility had a negative significant effect on efficiency. As shown in Table 2 the highest and lowest coefficient were related to the number of physicians per population in the fourth model and the number of midwives per population in third model respectively. In the fourth model, the number of physicians per population and the number of rural primary healthcare centers had a positive effect on efficiency, while the number of midwives per population had a negative effect on efficiency. As shown in Table 2, based on the log likelihood criteria, the fourth model was the best model to explain the factors affecting efficiency in PHCCs.

## Discussion

In this study, the efficiency scores of rural PHCCs and the factors affecting it were determined. Efficiency is one of the important indicators of productivity in order to compare the existing utilization with standard criterion and evaluate the performance of homogeneous and similar units.

The results of DEA analysis showed that the mean of total efficiency is low, about 0.6. One of the most important reasons for low efficiency can be related to that the health care system in Iran is treatment-based, Which treatment has priority over the prevention [31]. Most of health care resources go to hospitals and urban health care center and therefore can reduce the efficiency of rural primary health care centers. This result consistent with previous works. Many studies on DEA in Africa, Such as Ghana, Sierra Leone, and Burkina Faso have reported a high level of inefficiency in primary health care delivery, especially in rural areas [21, 22, 32, 33]. In Greece, the average technical efficiency score of inefficient centers was 0.57 [34].

In our study, the average of total efficiency of the centers had a fluctuating trend during the period of the study, but the average performance scores generally decreased in 2016, as compared with 2002. The highest and lowest average performance scores were observed in 2003 (0.78) and 2013 (0.56), respectively. In Rostami et al.’s study [17], which investigated the efficiency of rural health centers in Qazvin province between 2006 and 2010, it was found that although the technical efficiency of the centers improved over the period of the study, there was still a significant discrepancy between the current use and optimal use resources. The efficiency trend in this study was inconsistent with present study. This might be due to that the some surrogate outcomes such as percentages of screening for congenital hypothyroidism, percentages of iron supplementation in the first guidance school students, number of general practitioner’s visit, and number of specialist referral by the general physician was used as output in this study, while we used final outcome (child mortality rate) as a performance.

The finding of present research showed that Assadabad and Tuyserkan health centers had the highest efficiency scores and stability during 2002–2016. However, Hamadan had the minimum efficiency over this period. The efficiency score for rural primary health care centers in Hamadan was less than 0.66. The low efficiency of rural health centers in Hamadan district can be attributed to the high rate of migration, its position as the capital of the province, better access to hospitals and other public or private health centers. A study by Akzahli et al. [35] showed that 78% of Ghanaian primary health care facilities were technically inefficient. Kirigia et al.’s study [36] in 2001 also showed that 70% of primary health care clinics in South Africa were inefficient.

In Greece, the average technical efficiency score of inefficient centers was 0.57. More than half of the centers were operating with an efficiency score of above 0.9; in addition, 31% of the centers had poor efficiency (0.5–0.7) and 31.8% of the centers had a very poor efficiency (less than 0.5) [34].

The second part of this study investigated the factors affecting the efficiency of rural primary health centers. The results of Tobit regression indicated that the total number of physicians and the ratio of physician to population had a positive and significant relationship with the efficiency of rural primary health care centers. One possible explanation for this relationship could be that physicians are more knowledgeable than Behvarzs and other members of the family physician team about diagnose and treatment of diseases and strategies for quick referral procedure to hospitals. Thus, it can be concluded that physicians have a positive and critical role in the prevention and reduction of mortality among children under 1 year and 1 to 5 years of age in rural areas. Therefore, the increase in the number of physicians along with the diagnostic and therapeutic aspects will help to increase the access of people to services and improve the efficiency of health centers via reducing the mortality rate among children and infants. Various studies have shown that in children under the age of one, respiratory, cardiovascular, genitourinary system diseases, and in children aged 1 to 5 years, accidents and cancers are the most important causes of death [37–40].

Contrary to the number of physicians, the overall number of midwives and the ratio of midwives to population showed a negative significant relationship with the efficiency. The reason for this relationship is that in the rural primary health care centers, midwives’ duties and most services provided by midwives are highly overlapping with services provided by Behvarzs. Therefore, the increase in the number of midwives results in an increase in the inputs and thus has a negative effect on the efficiency of health houses.

Furthermore, fertility rate has a negative and significant relationship with efficiency. Various studies conducted around the world have shown that high fertility rates are directly associated with an increase in infant and child mortality [41, 42], that are consistent with the results of present study. With the increase in fertility rate, which are considered as the outputs, and since the inputs (number of health workers) remain constant, fertility rate is expected to have a negative effect on efficiency.

Regression analysis showed that the number of health houses in each district had a positive and significant relationship with the efficiency of rural primary health care centers. The increase in the number of health houses in a district improves people access to services provided by Behvarzs and other members of primary care teams; furthermore, it facilitates the ongoing monitoring and follow-up of services and ultimately improves health indicators. Facilitating access to services and improving health indicators, directly and indirectly reduces child mortality among those less than 1 year of age and those aged 1 to 5 years. In a study by Marshall et al. [21] which examined efficiency in Burkina Faso villages, it was found that poor utilization of health services facilities was the most important cause of inefficiency in rural health centers. The results of Tobit regression model in Marshall et al.’s study, which examined efficiency in Burkina Faso villages, showed that the prediction of efficiency scores was significantly associated with distance and socioeconomic variables [21]. In Al-Hassan et al.’s study [24] in Ghana, which examined the performance of public and private primary care centers, it was shown that none of the quality care proxies had a significant relationship with technical efficiency, but the geographical location of the centers and the type of ownership had a significant effect on the level of technical efficiency. This study has some limitations: First, we did not have access to all the data for all rural primary health care centers in Hamadan province. So that, the number of centers that included in the study were determined by data availability. Also, there was limited data on variables that would have helped to further explore predictors of inefficiency across facilities.

## Conclusion

The aim of this study was to investigate the efficiency of rural health houses and factors affecting their efficiency in Hamadan province. Except for the three districts (Asadabad, Bahar, and Tuyserkan), the health houses in most of districts did not operate at their maximum level of efficiency. It was found that the number of physicians, the number of midwives, the overall fertility rate, and the number of health houses in each district affected efficiency. According to the results, it is recommended to introduce the best units (best practices and benchmarks) to each of the inefficient units and executives in order to increase their efficiency and make plans for achieving the optimal performance in inefficient units. It is also recommended to control the overall fertility of rural women by conducting appropriate and educational programs by health workers. Because of the overlap of the services provided by midwives and Behvarzs, it is recommended to recruit a flexible number of midwives in rural health centers to cover more centers and increase the efficiency of health houses while adjusting the workforce.

## Data Availability

All data are available on reasonable request.
